# Improving the Quality and Quantity of HIV Data in the Middle East and North Africa: Key Challenges and Ways Forward

**DOI:** 10.15171/ijhpm.2016.112

**Published:** 2016-08-23

**Authors:** Mohammad Karamouzian, Navid Madani, Fardad Doroudi, Ali Akbar Haghdoost

**Affiliations:** ^1^HIV/STI Surveillance Research Center, and WHO Collaborating Center for HIV Surveillance, Institute for Futures Studies in Health, Kerman University of Medical Sciences, Kerman, Iran.; ^2^School of Population and Public Health, University of British Columbia, Vancouver, BC, Canada.; ^3^Department of Cancer Immunology and Virology, Dana-Farber Cancer Institute, Department of Global Health and Social Medicine, Harvard Medical School, Boston, MA, USA.; ^4^UNAIDS – The Joint United Nations Programme on HIV/AIDS (UNAIDS), Tehran, Iran.

**Keywords:** HIV, Data, Middle East and North Africa (MENA)

## Abstract

Although the HIV pandemic is witnessing a decline in the number of new infections in most regions of the world, the Middle East and North Africa (MENA) has a rapidly growing HIV problem. While generating HIV data has been consistently increasing since 2005, MENA’s contribution to the global HIV literature is just over 1% and the existing evidence often falls behind the academic standards. Several factors could be at play that contribute to the limited quantity and quality of HIV data in MENA. This editorial tries to explore and explain the barriers to collecting high-quality HIV data and generating precise estimates in MENA. These barriers include a number of logistic and socio-political challenges faced by researchers, public health officials, and policy-makers. Looking at successful regional HIV programs, we explore examples were policies have shifted and lessons could be learned in developing appropriate responses to HIV across the region.

The Middle East and North Africa (MENA)^ [1]^ covers an extensive region extending from Morocco to Iran and includes a number of low-, middle-, and high-income countries comprising 10% of the world’s 15-49 population.^1-3^ The socio-cultural, political, and logistical context of the region has created several barriers to addressing public health issues, effectively.^4^ In particular, adopting appropriate approaches to tackle sexually transmitted infections – HIV in particular – often leads to heated and controversial debates. Although the HIV pandemic is witnessing a decline in the number of new infections in most regions of the world, the MENA is one of the regions the world with a rapidly increasing HIV epidemic.^4^ Unlike sub-Saharan Africa where new annual HIV infections have declined by 41% since 2000, HIV transmission rates in the MENA rose by 17% during the same period; the second greatest growth in the world. Similarly, while the number of AIDS-related death have declined by 34% in sub-Saharan Africa since 2000, they indicate a 234% increase in the MENA; the greatest growth globally (Figure 1).^5,6^

**Figure 1:**
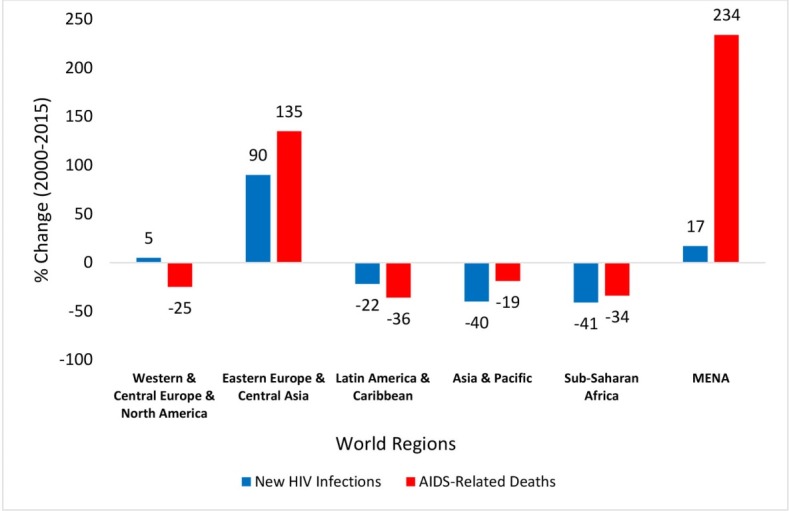
Percentage Change in the Estimated Annual Number of New HIV Infections and AIDS-related Death by World Region, 2000-2015.^5,6^

Despite the growing HIV problem in the MENA, its contribution to the global HIV literature is very limited.^7^ Moreover, as outlined in Figure 2, most of the contribution of MENA countries to HIV-related literature is coming from only a few countries (eg, 60% of the peer-reviewed publications indexed in PubMed are published in Iran [805 papers], Pakistan [464 papers], Egypt [196 papers], and Sudan [189 papers]). Is it the quality of HIV research that does not meet the required standards of academic journals and remains unpublished? Is this small contribution due to the poor human capital and a limited number of trained and experienced staff to conduct high-quality research and report their findings? Alternatively, are most MENA countries struggling with financing HIV research or is there a systematic politically- or ideologically-driven approach that limits generating high-quality HIV data? Are the ongoing and expanding conflicts in parts of the region making the HIV epidemic look like a “trivial” problem to policy-makers? Would strategies be shifted when policy-makers are provided with high-quality and solid evidence? In this editorial, we try to assess these questions and provide potential action steps in addressing the challenges around collecting high-quality and frequent HIV data in MENA countries.

**Figure 2:**
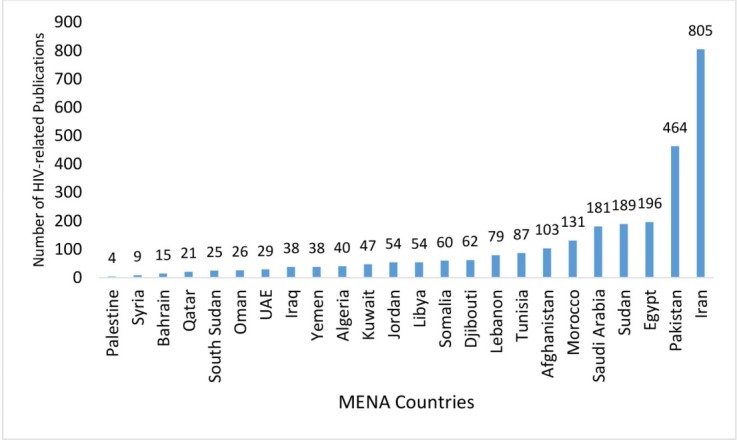
HIV-Related Publications in Different Countries Across the Middle East and North (MENA) Africa Region (Peer-Reviewed Publications Indexed in PubMed by July 2016).

## Limited HIV Data for Key Populations at Risk of HIV

In the MENA, the epidemic is believed to be driven by sexual and drug-related risky behaviours of key populations at risk of HIV. Main affected populations in the MENA are people who inject drugs (PWID), men who have sex with men (MSM), female sex workers (FSWs), and their sexual and injecting partners.^1,2,4^ In such concentrated epidemics, the estimates of HIV prevalence relies on the prevalence of HIV in each key at-risk population and the size of low- and high-risk populations. In the MENA region, however, data on HIV prevalence among key populations is unavailable in several low- and high-income countries (eg, Bahrain, Iraq, Kuwait, Libya, Oman, Qatar, Saudi Arabia, South Sudan, Syria, and the UAE).^4,8^ Moreover, the existing evidence do not portray the real picture of new HIV infections in the region and are prone to underestimation biases due to their reliance on the poor, infrequent, and non-representative (eg, facility-based) surveillance systems within the Ministries of Health.^4^ Indeed, only three countries (ie, Djibouti, Iran, and Morocco) in the MENA have been reported to have a relatively stable and functioning HIV surveillance system.^4,8,9^ Moreover, no country has managed to have more than three rounds of integrated bio-behavioral surveillance surveys (IBBS) among key populations. Egypt, Lebanon, and Tunisia are the only three countries to have conducted IBBS in all three key populations (ie, FSWs, MSM, PWID).^4,8^ Additionally, several key populations (eg, FSWs, MSM) continue to be criminalized and are excluded from surveillance programs due to several socio-political sensitivities.^4,7^ Other populations such as foreign migrant workers, military personnel, refugees, street children, and internally displaced individuals are also often overlooked or missed in monitoring and evaluation sectors.^4,8^ Therefore, great uncertainties exist around estimating the size of key populations at risk of HIV in the MENA, MSM, and FSWs in particular.^1,10^

## Limited HIV Data for the General Population

A significant problem affecting the completeness, validity, and reliability of HIV data in the MENA is the absence of precise or evidence-based population data. The region has been facing several conflicts, wars, and uprisings in the past few years. The recent ongoing forced displacement of civilians combined with the chaotic economic and socio-political situations has created one of the worst humanitarian crises in the past century. Thousands of people have been killed, wounded, or forced into migrations inside and outside their countries. In the last 10 years, several countries have been directly and indirectly involved in conflicts and civil wars in the MENA (eg, Bahrain, Egypt, Iraq, Lebanon, Palestine, Saudi Arabia, Somalia, Sudan, Syria, and Yemen).^11^ In Syria for example, approximately 8.7 million people are estimated to be displaced internally, and 4.8 million to have left Syria by the end of 2015.^12^ In Iraq, as many as 3.9 million Iraqis were internally displaced by mid-2015.^12^ Therefore, estimating the size of the population or defining the population – which are key indicators in calculating HIV rates – remain very challenging and result in impaired quality of estimates.^13,14^ Furthermore, forced displacement may contribute to the vulnerability of refugees to HIV; survival sex practices in exchange for food and resources (eg, housing, jobs) have been reported from refugees across various settings including Lebanon, Jordan, and Cyprus.^15-18^

Given the challenges around reaching precise estimates, adjustment values for the plausibility bounds in most countries in the MENA are obtained through consultancies with national HIV experts, which would have implications for the validity and reliability of the estimates and is susceptible to some limitations, misinterpretations, and subjectivities. Indeed, the evidence on HIV data points to a wide discrepancy between estimated cases of HIV and official national statistics; leading to contradictory interpretations of the course of the HIV epidemic in the region. By the end of 2015, the registered number of people living with HIV (PLHIV) across all MENA countries were far less than the estimated national numbers.^6^ For example, while Iran’s Ministry of Health reported about 31 000 registered PLHIV cases, estimates were suggesting 73 000 PLHIV to be living in Iran.^19,20^ Moreover, figures in some settings are incredibly low and raise concerns about their reliability. For example, in Egypt with a population of 82 million, the official number of people living with HIV (PLHIV) was estimated at around 11 000^21^; a figure much lower than that of Sudan (around 56 000 PLHIV in a population of 38 million)^22^ or Morocco (around 24 000 PLHIV in a population of 33 million).^23^

## Social and Political Will for a Change: Easier Said Than Done

The beginning phases of HIV discovery was overshadowed with the “not in our land” policy, and the MENA was assumed to be socio-culturally “immune” to the HIV epidemic. HIV was assumed to be less prevalent in the MENA due to the dominant practice of Islamic rituals (eg, circumcision and ablution) and conservative socio-cultural settings (eg, stigmatizing and prohibiting alcohol consumption, substance abuse, pre/extramarital sex, and homosexuality).^24,25^ There is a wide perception that the socio-cultural and political sensitivities associated with HIV research in the MENA as well as structural-level HIV-related stigma, may restrict access to HIV-related data and create barriers in conducting HIV research in the region.^26^

Indeed, making decisions to address HIV in concentrated epidemics – where key populations are at the core of the preventative interventions – could be a real challenge in contexts where the existence of most key populations (eg, MSM, FSWs) is denied. Therefore, the political cost of allocating public funds to deal with their needs and problems could be concerning or “politically unsafe” for many public health officials; leading to underfunding of HIV research at the national level.^1,4,25-27^ In 2010 for example, per capita domestic governmental spending on HIV ranged between less than five cents in the poorest to just over $1 in the richest countries in the region.^1,28^ This placed the MENA at the bottom of the world regions in public fund spending on HIV.^1,28^ As a result, most of the quality research conducted so far has mainly been funded by international funders such as the Global Fund to Fight AIDS, tuberculosis (TB), and Malaria (GFATM) which represents over 70% of the support for HIV/AIDS in the MENA.^1,29^ The US National Institutes of Health (NIH), Bethesda, MD, USA is another international funder that has recognized the need to scale up HIV research in the MENA and supported novel HIV research in Egypt, Lebanon, Morocco, and Tunisia. However, recent revisions made to the financing criteria of GFATM have raised concerns about the future of these sources of support for some countries in the MENA (eg, Tunisia, Morocco).^1^ Furthermore, several middle-income countries in the MENA are ineligible for other international funds (eg, US President’s Emergency Plan for AIDS Relief - PEPFAR or bilateral support of other donor nations).^1,8,30^ Regardless, such international investments have, indeed, led to improvements in the quality of HIV research by training national experts on state-of-the-art approaches for community-based HIV surveillance surveys among key populations (eg, respondent driven sampling approaches) and hence resulted in an increase in the improved visibility of HIV research from the MENA in high impact peer-reviewed journals.^7,26^ Nonetheless, despite the increasing body of evidence on HIV research in the MENA since 2005, it is still limited (ie, less than 2% of the global HIV literature) and several findings are unpublished, and large databases remain unanalyzed and inaccessible.^7,29^

Fortunately, despite the undeniable existing socio-cultural barriers, most regional public health officials and policy-makers have started to show interest in addressing the issue. The development of the Arab AIDS Strategy in 2014 is a testimony to the socio-political will of 22 Arab countries in *working towards an AIDS-Free Generation in Arab countries*. Moreover, the Regional Arab Network against AIDS’s Memorandum of Agreement with these Arab States about the role of civil society in implementing the Arab AIDS Strategy is a step forward in recognizing the important role of civil society in addressing the epidemic in the region.^18^ Although these initiatives are impressive and set the ground for sharing best practices across the region, some believe that there is still room for improvement in the HIV prevention efforts of certain members of the Arab League in reaching the goals of the Arab AIDS strategy.^18^

## Lessons Learned and Path Forward

While the MENA region is facing a rapid increase in the number of PLHIV across different sub-populations, it has a unique and real window of opportunity to optimize the output of interventions and avoid the future burden of HIV on its population (Table). Considering significant degrees of uncertainty around the registered and estimated HIV cases, it is critical to create precise estimates that provide the governments with a real picture of the problem. Indeed, when concrete and quality HIV data are presented to policy-makers in the MENA, they could lead to policy shifts. Several regional successful examples of implementation of effective risk-group size estimations, mapping, and HIV surveillance among key populations exist that could be scaled up and utilized across other MENA countries. In other words, success stories and promising culturally appropriate interventions that help move away from misperceptions to a sound evidence-based approach do exist. For example, Iran, Morocco, and Lebanon have successfully expanded opioid substitution therapy (OST) programs within their communities. Moreover, needle and syringe programs (NSPs) have helped increase safe injection practices to 70% among PWID in Iran, Lebanon, Morocco, and Tunisia.^6,9,19,31,32^ Interventions aimed at improving the sexual health of MSM have also shown promise where services take on a rights-based approach to HIV prevention and when civil society is enabled to provide support for the community. For example, 75% of MSM in Lebanon have reported consistent condom use and knowledge of their HIV status. HIV prevention efforts among FSWs have also demonstrated success with condom use rates as high as 80% among FSWs in Algeria and Lebanon.^2,6,33^

**Table:** Challenges in Generating High-Quality and Frequent HIV Data in the MENA and Action Steps Needed to Improve the Quality and Quantity of HIV Data in the MENA

**Challenges**	**Action Steps**
Limited studies on key populations at risk of HIV	• Acknowledging the burden of the epidemic and recognizing key populations as a reality • Conduct population size estimation studies
Exclusion of foreign migrant workers, military personnel, refugees, and street children from HIV surveillance programs	• Commitment to fund and conduct consistent and frequent surveillance surveys
Ongoing conflicts leading to forced displacement of the population	• Scaling up outreach programs to reach the refugee and displaced population
Policy-making dilemmas in funding HIV research	• Combating HIV-related stigma and discrimination at individual, community, and structural-levels • Coordination between ministries of health, religious leaders, and criminal justice system
Limited national research budgets	• Further financial support from international organizations as well as from wealthy countries in MENA not facing political unrests • Establishment of a regional funding resource to share the financial burden of responding to HIV
Lack of publicly available databases	• Developing regional data sharing policies and publicly available databases to improve data access for foreign researchers and promote international and regional collaborations
Limited human capital	• Training a generation of young local or regional HIV researchers

Abbreviation‏: MENA, Middle East and North.

As these successful experiences are not necessarily shared across MENA countries, establishing and supporting a regional network of HIV research where countries and researchers could share the details and challenges of implementing these successful regional experiences (eg, through regional workshops, meetings, or collaborations) could be highly beneficial. The Global Network of Researchers on HIV/AIDS in the Middle East and North Africa Region (GNR-MENA) which is a forum for scientific exchange, debate and networking for researchers involved or interested in the HIV response in the region, has served as a platform for knowledge transfer; however, the voluntary nature of the executive positions at GNR-MENA may have limited its capacity for effective action. Furthermore, developing regional data sharing policies and publicly available databases could improve data access for potential foreign researchers and promote regional and international collaborations; partnerships that could help improve the quality of research, help build research capacities and increase the visibility of HIV-related publications in the MENA. While the MENA’s young population could fuel the epidemic, youth could, indeed, serve as an opportunity for the human capital development and training a generation of young local HIV scientists could make significant contributions to high-quality data generation in the MENA. Moreover, establishing regional journals that focus on HIV surveillance in the region could also be a viable approach in promoting HIV research and sharing experiences across the region.

Nonetheless, high-quality HIV research is not feasible in the MENA as long as profound individual-, organizational-, and structural-level stigmas are present at different layers of the society. To combat the profound systemic multi-level stigmas around HIV and impact public opinion, multi-sectoral and comprehensive upstream policies should be developed and implemented. All informed stakeholders from researchers to policy-makers and religious leaders, should proactively collaborate, set their differences aside, and further engage in exchanging ideas in a scientific and evidence-based environment to help develop successful HIV programs in the MENA. Lessons learned during the implementation of Kerman HIV-Friendly City – an ongoing population-level intervention focused on creating an HIV-stigma free city in Iran – are a testimony to the feasibility of reaching these goals and could provide insight into the implementation of anti-stigma interventions across the region.

Furthermore, considering the global economic crisis and resources drying up, and the continuing conflicts across the region, MENA countries need to reconsider their funding policies towards HIV/AIDS research and preventative efforts at national and regional levels; the most cost-effective investments should be identified. Countries facing budget constraints in spending on HIV research and prevention efforts, however, should be offered financial support from other regional and international organizations. In particular, some countries in the MENA have the wealth, capacity, and resources tto taking HIV spending to another level both inside and outside their countries. Wealthy countries in the MENA not facing political unrests could contribute further by establishing a regional funding resource to share the financial burden of responding to HIV and help further scale up outreach programs reach people living in refugee camps and collect data on the status of PLHIV and vulnerable population.^1,18^ MENA’s leaders and policy-makers should be aware and act upon the fact that HIV will not stop at countries’ borders and could affect all countries in the region.

## Acknowledgements

Authors are grateful to Dr. Peter Ghys at the Strategic Information and Evaluation department of UNAIDS for his scientific and editorial input. The views and opinions expressed in this paper are those of the authors and not of UNAIDS.

## Ethical issues

Not applicable.

## Competing interests

The authors declare that they have no competing interests.

## Authors’ contributions

Concepts, Design, and manuscript editing: MK, NM, FD, AAH; Literature search and manuscript drafting: MK; Data acquisition and data analysis: MK; All of the authors made substantial suggestions for the revisions of the manuscript and approved the final submitted version of the paper.

## Authors’ affiliations

^1^HIV/STI Surveillance Research Center, and WHO Collaborating Center for HIV Surveillance, Institute for Futures Studies in Health, Kerman University of Medical Sciences, Kerman, Iran. ^2^School of Population and Public Health, University of British Columbia, Vancouver, BC, Canada. ^3^Department of Cancer Immunology and Virology, Dana-Farber Cancer Institute, Department of Global Health and Social Medicine, Harvard Medical School, Boston, MA, USA. ^4^UNAIDS – The Joint United Nations Programme on HIV/AIDS (UNAIDS), Tehran, Iran.

## Endnote


[1] For the purpose of this editorial, MENA is defined based on the MENA definitions of UNAIDS and the Eastern Mediterranean Regional Office of the World Health Organization (WHO/EMRO) and the following countries are considered: Afghanistan, Algeria, Bahrain, Djibouti, Egypt, Iran, Iraq, Jordan, Kuwait, Lebanon, Libya, Morocco, Oman, Pakistan, Palestine, Qatar, Saudi Arabia, Somalia, Sudan, South Sudan, Syria, Tunisia, United Arab Emirates (UAE), and Yemen.^3,4^

